# T31. TEN YEAR CLINICAL FOLLOW-UP OF YOUTH WITH EARLY ONSET PSYCHOSIS

**DOI:** 10.1093/schbul/sby016.307

**Published:** 2018-04-01

**Authors:** Sugranyes Gisela, Elena De la Serna, Marina Redondo, Daniel Ilzarbe, Josefina Castro-Fornieles, Inmaculada Baeza

**Affiliations:** 1 Hospital Clínic of Barcelona; 2 Centro De Investigación Biomédica En Red De Salud Mental, CIBERSAM; 3 Hospital Clínic de Barcelona, Centro de Investigación Biomédica en Red de Salud Mental, CIBERSAM

## Abstract

**Background:**

Early onset psychosis (onset prior to age 18) may be associated with poorer long-term outcomes than when onset takes place in adulthood. Studies so far prospectively following youth with early onset psychosis have suggested that only 20% of patients experienced a “good” outcome, and that nearly 50% experience a “poor” outcome. However, the major body of evidence so far has focused on cohorts recruited over 20 years ago, while a recent review (Clemmensen 2012) has noted that outcomes may be more favourable in more recent studies.

We set out to update the evidence by prospectively assessing youth with a first episode of early onset psychosis after a 10 year follow-up period with clinical and functional measures.

**Methods:**

Patients were recruited from a child and adolescent psychiatry unit at a university hospital in Barcelona, Spain between 2003 and 2008. Inclusion criteria were: age 7 to 17 years and presence of positive psychotic symptoms of less than 6 months duration. Exclusion criteria: presence of a concomitant Axis I disorder that could account for the psychotic symptoms (e.g., substance abuse, autistic spectrum disorders, post-traumatic stress disorder, or acute stress disorder); learning disability according to DSM-IV criteria; neurological disorders. Occasional substance use was not an exclusion criterion. A sample of age matched healthy controls was recruited from the same geographical area. Controls had the same exclusion criteria in addition to no family history of psychotic disorders in first degree relatives. All participants were assessed with clinical (K-SADS, PANSS, GAF, CGI) measures by a psychologist and psychiatrist with experience with child and adolescent population at baseline and 10 year follow-up.

**Results:**

Sixty-nine patients and thirty-one controls were assessed at baseline; 36 patients (52%) and 24 controls (77%) were re-assessed at ten year follow-up. There were no differences in age (M=26.4 SD=1.4 vs. M=26.0 SD=2.0; t=.84; p=.41) or gender (47% vs. 46% females, Chi square=.011; p=.92). We were unable to locate 9 patients (13%) and 4 controls (12.5%). Two patients were deceased at follow-up (one committed suicide; one from metastasic cancer), one patient was in prison, and the rest declined participation. Patients who attended follow-up had trend-level baseline poorer functioning (17.85 vs. 22.4, p=.065), and significantly greater baseline clinical severity 6.44 vs 6.0, p=.017) than those who did not. Diagnoses at baseline were as follows: n=16 (23.2%) schizophrenia; n=20 (29%) affective disorders with psychotic symptoms; n=29 (42%) psychosis not otherwise specified. At follow-up: n=18 (50%) schizophrenia, n=11 (30.6%) affective disorders, n=1 (2.8%) personality disorder; n=1 (2.8%) eating disorder and n=5 (13.9%) had no psychiatric diagnosis. Twenty patients (58.8%) had been hospitalized during the follow-up period and thirty (88.2%) were currently receiving at least one antipsychotic drug. Seven patients (19.4%) were categorised as “poor outcome” (GAF < 50), 18 patients (50%) as “moderate” outcome (GAF 51–70) and 9 patients (26.5%) as “good outcome” (GAF >70). Seven patients (19.4%) were in full time employment and five (13.9%) were in full time education. The rest were either unemployed or working part time or in an assisted setting.

**Discussion:**

Early onset psychosis is associated with poor long term outcomes in a portion of patients, although current functional outcomes in these patients may be comparatively better in relation to data from historical reports (Clemmensen 2012). Factors related to improved healthcare services, such as reduction in duration of untreated psychosis and new treatment modalities may potentially underlie these differences.

